# Dual-Branch Point Cloud Semantic Segmentation: An EMA-Based Teacher–Student Collaborative Learning Framework

**DOI:** 10.3390/s26020450

**Published:** 2026-01-09

**Authors:** Xiaoying Zhang, Yu Hu, Yuzhuo Li, Zhoucan Nan, Qian Yu

**Affiliations:** 1 School of Mechanical Engineering, Jiangsu University of Technology, Changzhou 213001, China; 2023655321@smail.jstu.edu.cn (X.Z.); 2023655081@smail.jstu.edu.cn (Y.H.); 2023655038@smail.jstu.edu.cn (Y.L.); 2024655178@smail.jstu.edu.cn (Z.N.); 2 School of Computer Engineering, Jiangsu University of Technology, Changzhou 213001, China

**Keywords:** weak supervision, point-cloud semantic segmentation, data augmentation, deep learning

## Abstract

Point cloud semantic segmentation remains challenging under extremely low annotation budgets due to inefficient utilization of sparse labels and sensitivity to data augmentation noise. To address this, we propose a dual-branch consistency learning (DBCL) framework featuring an EMA teacher for semi-supervised point cloud segmentation. Our core innovation lies in a unified consistency regularization scheme that enforces prediction-level alignment via JS divergence and feature-level contrastive learning, while a geometry-aware Laplacian smoothing term preserves local structural consistency. Extensive experiments demonstrate that DBCL achieves 68.56% mIoU on S3DIS with only 0.1% labels, outperforming existing semi-supervised methods and even matching some fully supervised baselines.

## 1. Introduction

Point cloud semantic segmentation is a critical task in computer vision that involves assigning semantic labels to individual points within a three-dimensional (3D) point cloud. This methodology has garnered considerable attention due to its wide-ranging applications, particularly in navigation and perception for autonomous vehicles, robotics, and virtual reality environments.

Point cloud data is typically collected using light detection and ranging, a crucial technology that enables self-driving vehicles in their environmental detection capabilities [1]. Semantic segmentation methods for point clouds have advanced significantly through fully supervised learning approaches. However, PointNet [2] faces substantial computational challenges and burdens when processing raw point cloud data directly, particularly when dealing with large-scale datasets, limiting its suitability for real-time application requirements. In contrast, RandLA-Net [3] enhances efficiency by using random downsampling to select a subset of points from the input point cloud for training. While this approach supports real-time performance, it can compromise segmentation accuracy in more complex scenarios. Both approaches depend heavily on extensive point cloud annotations. For instance, labeling complex areas in SemanticKITTI [4], such as residential neighborhoods, is highly labor-intensive, significantly driving up annotation costs.

To reduce time and labor costs, weakly supervised learning methods for point clouds have emerged, training networks with only a small subset of labeled points. By relying on sparse or minimal annotations, these approaches significantly reduce annotation efforts and time expenses. Recent studies [5,6,7,8,9] have explored learning from weakly annotated point clouds, using the consistency constraint technique [10] to improve segmentation accuracy. This approach reduces the gap between predicted and actual point clouds by comparing an unlabeled data point with its perturbed version [11]. Although this approach improves point cloud segmentation and reduces labeling costs, it remains limited in capturing the full complexity of point cloud data. Dependence on consistency constraints and a small set of labeled points is insufficient for extracting the rich contextual information embedded in the point cloud.

Over the past two years, the perturbation self-distillation (PSD) [12] framework has emerged to address the aforementioned challenges. Drawing on self-supervised learning principles, PSD generates perturbation branches aimed at enhancing predictive consistency between these branches to strengthen consistency between them and the original branches. By integrating an auxiliary supervision mechanism, it effectively captures the graph topology of the entire point cloud, thereby enabling better information transfer between labeled and unlabeled points. Despite achieving significant average improvements, this method has several limitations. First, relying on a single-layer RandLA-Net [3] for point cloud training can result in the loss of intricate details, reducing the model’s ability to accurately capture point cloud features. Second, insufficient supervisory information may restrict the model’s adaptability to varied point cloud structures. Lastly, the method’s success heavily depends on the network’s ability to understand point cloud features and acquire effective supervisory information, both of which are crucial for ensuring high-quality point cloud generation.

To address the aforementioned challenges, this study proposes a novel heterogeneous dual-branch consistency learning framework that enhances semantic segmentation performance under limited supervision. Unlike conventional mean teacher approaches [13] with identical architectures, our method employs structurally asymmetric encoders, a deep student network, and a shallower-but-wider teacher network, where the teacher’s parameters are updated via an exponential moving average (EMA) with cross-architectural variable mapping. This design allows the teacher to act as a stabilized, distilled version of the student, providing consistent target signals while preserving essential geometric details. Beyond the standard prediction-level consistency, we introduce multi-hierarchical consistency constraints that simultaneously regularize predictions, features, and local structures.

To generate diverse yet geometrically meaningful perturbations, we propose a structure-aware augmentation module that incorporates four distinct operations, mirroring, rotation, coordinate jittering, and regionwise masking, applied randomly during training. Importantly, the augmentation process is weighted by a learned channel attention mechanism, allowing the model to emphasize informative feature channels. Furthermore, an explicit Structure Relation Context (SR-Context) module is embedded to encode local geometric relationships, providing an inductive bias that is particularly beneficial for outdoor and medical point clouds with strong structural patterns. Beyond the standard prediction-level consistency, we introduce multi-hierarchical consistency constraints that simultaneously regularize predictions, features, and local structures.

In summary, the main contributions of this study are outlined as follows:

(1) We propose a structurally asymmetric dual-branch design featuring a deep student encoder and a shallow-but-wide teacher encoder. The teacher’s parameters are updated via a cross-architectural exponential moving average (EMA) with tailored variable mapping, which stabilizes training and preserves fine geometric details often lost in homogeneous setups.

(2) We introduce a unified regularization scheme that enforces agreement at three complementary levels: prediction consistency through Jensen-Shannon divergence, feature-level consistency via contrastive regularization (CR loss) that clusters same-class embeddings, and local structural consistency through a novel Laplacian smoothing loss that encourages similar predictions among neighboring points. This multi-level approach is systematically implemented in the loss function.

(3) We design a structure-sensitive augmentation module that combines mirroring, rotation, jittering, and regionwise masking, weighted by a learnable channel attention mechanism (data augment). Furthermore, an explicit Structure Relation Context (SR-Context) module captures local geometric relationships via edge convolution, providing a strong inductive bias for segmenting complex outdoor and medical point clouds.

(4) We conduct rigorous evaluations on multiple large-scale benchmarks (including S3DIS and Toronto-3D) under extremely low-label regimes (e.g., 0.1% labels). Our framework not only outperforms existing weakly and semi-supervised methods but also matches or surpasses several fully supervised baselines, demonstrating its effectiveness and robustness.

The rest of this paper is organized as follows: Section 2 reviews related work on semi-supervised, fully supervised, and unsupervised point cloud segmentation. Section 3 details our proposed heterogeneous dual-branch framework, including the EMA teacher mechanism, structure-aware enhancement, the SR-Context module, and the design of the loss functions. Section 4 presents extensive experiments on benchmarks such as S3DIS and Toronto-3D, including comparisons with state-of-the-art methods, ablation studies, and qualitative analyses. Finally, Section 5 concludes this paper and discusses potential directions for future research.

## 2. Related Work

### 2.1. Fully Supervised Point Cloud Semantic Segmentation

The ongoing advancements in deep learning and 3D sensing technologies have greatly enhanced the accuracy of semantic segmentation in 3D point clouds. Traditionally, fully supervised semantic segmentation approaches fall into three main categories: point-based methods [2,3,14,15], voxel-based methods [16,17,18], and hybrid methods [19,20]. PointNet [2] is a representative point-based approach that utilizes a shared multi-layer perceptron to learn features at the individual point level, followed by maximum pooling to aggregate global features. While it effectively handles the unordered characteristics of point clouds, its independent learning of each point feature limits its ability to capture complex local features.

Despite its limitation, PointNet [2] marks a foundational breakthrough in point cloud semantic segmentation. In contrast, PointNet++ [14] incorporates the farthest point sampling technique [21], which begins with an initial point and iteratively selects the most distant points to ensure diverse coverage. This sampling strategy, integrated into the PointNet [2] architecture, enhances the extraction of additional local geometric features. Although PointNet++ effectively mitigates issues related to density inhomogeneity within point clouds, it still struggles to capture fine-grained features.

In advanced point cloud applications, RandLA-Net [3] introduces a novel local feature aggregation module (LFAM), which improves the extraction of local geometric information by applying feature weighting and aggregating features across various scales.

In the voxel-based methodology [16,17,18], SEGCloud [16] effectively reduces computational complexity and achieves accurate segmentation of dense point clouds by leveraging downsampling and fractional interpolation. To further refine the segmentation outcomes, particularly in boundary regions, a conditional random field is applied as a postprocessing step. In contrast, PointGrid [17] enforces a fixed number of sampling points within each voxel, enabling the construction of higher-order local approximation functions that enhance the representation of local geometric details. This approach overcomes the limitations of SEGCloud [16] with large-scale scene segmentation and high computational cost. However, it may still lose detailed information within the point cloud, especially when using low voxel resolutions. Hybrid segmentation methodologies [19,20] combine multiple point cloud representations, such as points, voxels, and grids, with multi-resolution or multi-scale learning frameworks. This strategy addresses the shortcomings of using a single representation and improves semantic segmentation by integrating these diverse inputs within a unified network architecture or through the joint co-optimization of multiple modules. The fully convolutional point network (FCPN) [19] converts the original point cloud data into structured voxels within the network. This strategy enhances data storage efficiency while minimizing spatial redundancy and reducing the model’s storage requirements.

Although these methodologies have shown strong results on certain datasets, fully labeling point clouds is still time-consuming and costly. Furthermore, these methodologies often lose critical edge information during the training phase, which reduces fine-grained segmentation accuracy. To address these challenges, our proposed network achieves reliable segmentation outcomes using only a minimal fraction (e.g., 0.1%) of labeled point clouds for training. This approach not only cuts down the labeling time but also improves overall efficiency. Moreover, the two-layer network is specifically designed to better capture complex point cloud features, achieving an optimal balance between processing efficiency and segmentation performance.

### 2.2. Weakly Supervised Point Cloud Semantic Segmentation

With the growing availability of large-scale point cloud datasets containing billions of points, the associated costs of comprehensive labeling have driven increased interest in weakly supervised methodologies for point cloud segmentation tasks. Xu and Li et al. [9] introduced a multibranch supervision approach combined with a smoothing branch to improve small-scale point cloud representation. However, their use of a parameter-free graph for postprocessing is nonlearnable and demands significant GPU memory consumption when applied to large-scale point clouds. In contrast, Zhang, Yachao et al. [22] proposed a transfer learning-based approach to improve weakly supervised point cloud segmentation, relying on an additional dataset to leverage prior knowledge. Acquiring and applying knowledge to weakly supervised segmentation tasks remains a considerable challenge, primarily due to the labor-intensive pretraining process and the need for large datasets. Xu et al. [21] proposed a weakly supervised methodology based on labeling only a small number of points. However, this approach struggles with large-scale point clouds under sparse annotations, as it lacks learnable topological relationships and incurs high computational requirements associated with the use of the Laplacian matrix. Additionally, the method assumes uniformly distributed annotations across the point cloud, which may not reflect real-world scenarios. In contrast, the PSD framework proposed by Zhang, Yachao et al. [12] heavily depends on the contextual cues within the training data and employs a single-layer network which ultimately yields a suboptimal generalization of the model.

To address these challenges, we have proposed a dual-branch network capable of effectively extracting semantic feature information from point clouds, making them well suited for large-scale point cloud applications. Additionally, we have introduced a supplementary data enhancement module that strengthens feature representation. Unlike Zhang, Yachao et al. [22], who depend on an external dataset for pretraining, our approach focuses on leveraging the inherent supervisory signals within the data itself. By introducing diverse perturbations to the point cloud, the network learns to capture information across multiple scales, leading to more accurate segmentation. The unique prediction outputs—generated for the point clouds—are then perturbed and refined through a loss function, injecting additional supervisory signals to enhance the precision of weakly supervised segmentation.

### 2.3. Unsupervised Learning

Unsupervised learning methodologies aim to reduce reliance on manual labeling by employing techniques such as self-supervised learning and clustering. Applied to fully unlabeled point cloud sequences, these approaches extract semantic cues directly from the data, enabling effective point cloud segmentation without labeled input.

The OGC [21] framework offers a generalized, unsupervised approach to 3D object segmentation, capable of identifying multiple objects within a given dataset. Trained entirely on unlabeled point cloud sequences, it learns to segment objects by analyzing motion cues inherent in the data. After training, OGC [21] can perform object segmentation directly on a single-frame point cloud. A key strength of the OGC [21] framework lies in its use of specially designed loss functions that leverage motion information to generate supervisory signals, ensuring accurate segmentation while preserving the geometric integrity of the objects in motion. OGC [21] processes a singular point cloud input and produces multiple object masks in a single forward pass. Despite the effective use of motion constraints in the [21] framework to enhance point cloud segmentation training, its performance is considerably reduced when applied to stationary objects such as trees, traffic lights, and buildings, where segmentation accuracy remains limited.

To address the identified challenges, a self-supervised masking strategy is integrated into the perturbation component of our network. This strategy leverages semantic features from labeled and unlabeled point clouds, using information extracted from the labeled point clouds to provide effective supervision for the unlabeled data. Consequently, this approach enriches contextual understanding beyond the labeled point clouds, thereby optimizing the training process of the network. It not only boosts the model’s generalization abilities but also introduces a novel conceptual framework.

## 3. Methodology

### 3.1. Overall Network Model Architecture

**Figure 1:**
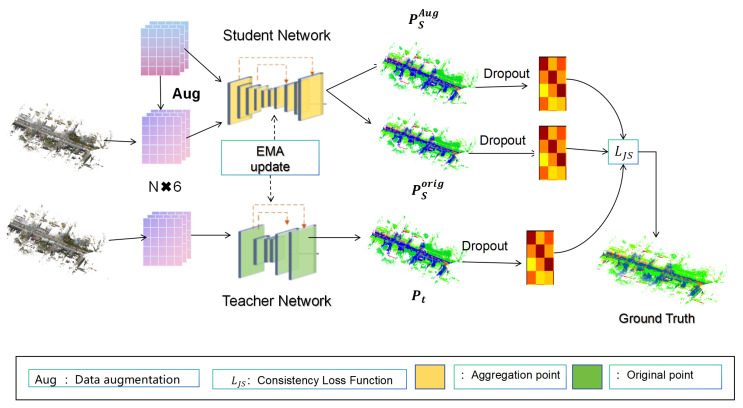
The primary structure of the network is outlined as follows: Given a point cloud P, the points are fed into the student network, which utilizes an encoder–decoder architecture to generate predicted point clouds $P_{s}^{orig}$ and $P_{s}^{Aug}$, respectively. Simultaneously, a three-layer teacher network outputs a predicted point cloud ($p_{t}$), with its parameters updated via an exponential moving average (EMA) mechanism. Finally, consistency functions $L_{JS}$ are applied between $P_{s}^{Aug}$ and both $P_{s}^{orig}$ and $p_{t}$ to enforce effective consistency regularization between the student and teacher predictions.

The overall network framework diagram is shown in Figure 1. Point cloud data, denoted as P, typically contains N points, each defined by spatial coordinates (x, y, z) and color attributes (r, g, b). The dual-branch consistency learning network proposed in this study employs an enhanced hierarchical encoder–decoder architecture with an exponential moving average (EMA) teacher mechanism. This network consists of two main synergistic components. The traditional mean teacher [13] employs identical network architectures for both student and teacher models. The teacher’s parameters are a direct exponential moving average (EMA) of the student’s parameters, where architectures are symmetric. We implement structurally asymmetric encoders while maintaining parameter update linkage through a novel variable-mapping EMA scheme. Our architectural divergence introduces functional specialization: the student explores complex feature representations while the teacher maintains a stabilized, distilled feature space. The EMA update propagates not just parameters but architecturally transformed knowledge from deep to shallow representations.

In the first component, the original point cloud undergoes comprehensive data augmentation. The traditional mean teacher applies generic augmentations (flips, rotations, noise addition). We introduce regionwise masking and channel attention mechanisms. Both the original and augmented point clouds are processed through a multi-scale feature extraction pipeline comprising dilated residual blocks with relative position encoding and attention-based pooling operations. The encoder utilizes random sampling and neighborhood aggregation to capture hierarchical features, while the decoder employs nearest interpolation to recover spatial resolution. In the second component, the network implements a student–teacher paradigm where the student network processes augmented inputs and the teacher network, updated via EMA from student parameters, provides stable supervisory signals. Consistency constraints are enforced through a multi-objective optimization framework combining cross-entropy loss, Lovasz loss, JS divergence between student and teacher predictions, contrastive regularization for feature embedding alignment, and Laplacian smoothing for spatial coherence. The traditional mean teacher enforces consistency through a single loss term (typically MSE or KL divergence) between student and teacher predictions.We implement a multi-tier consistency framework. This comprehensive loss strategy enables effective knowledge transfer between labeled and unlabeled data while preserving structural integrity across varying point densities. The proposed framework enhances semantic feature extraction through structural context preservation modules that explicitly model local geometric relationships and adaptive feature aggregation, significantly improving the network’s capability to capture both fine-grained details and complex contextual information in 3D scenes.

### 3.2. Point Cloud Augmentation Branching

To enhance model robustness and generalization capabilities while extracting more stable semantic features from limited labeled data, controlled perturbations are applied to the input data or features, thereby forming a perturbation branch. Various perturbation operations are first applied to the original point cloud, including geometric transformations (rotation and scaling), noise injection, and mirroring. These augmentations help ensure that the model’s predictions stay consistent across different perturbations, thereby mitigating the sensitivity of the point cloud to noise. Furthermore, a self-supervised region masking strategy is employed, which systematically masks portions of the point cloud in geometric space. This approach generates extra supervisory signals during training by leveraging the data’s inherent structural or semantic information. Training data selection is performed probabilistically, helping the model learn more robust semantic features.

Before the training process begins, the original point cloud is mapped into feature dimensions using a fully connected layer, preparing it for further network processing. The input data for the current layer of the original point cloud is characterized by the coordinates $\left(x_{i=1}^{n},y_{i=1}^{n},z_{i=1}^{n}\right)$, where $N_{i=1}^{n}$ represents the neighbor index for the current layer, and $S_{i=1}^{n}$ signifies the downsampling index for the same layer. Data augmentation employs several techniques, including mirror transformation, axis rotation, jittering, and self-supervised masking strategy. Additionally, the module selects these enhancements randomly, following a probability distribution aligned with predetermined criteria.

(1) $$P\left(k\right)=\frac{1}{4},k\in \left\{0,1,2,3\right\}$$

The mirror transformation, as detailed in reference [23], is a rigid body reflection, a type of linear transformation that symmetrically reflects the point cloud along the *Y*-axis, which can be mathematically expressed as $P^{r}={\left(x_{i=1}^{n},-y_{i=1}^{n},z_{i=1}^{n}\right)}^{T}$. This operation maintains the topological properties of the object and necessitates augmenting the model to achieve invariance to reflective symmetry.

The random rotation matrix, described in reference [24], is essential for 3D point cloud data. Its primary role is to enhance model robustness and generalization abilities by applying diverse geometric transformations. This method implicitly enlarges the training dataset and decreases the model’s dependence on the original orientation distribution. In our approach, we utilize a random rotation matrix to rotate the point cloud coordinates around the *Y*-axis.

(2) $$R_{y}\left(\theta \right)=\left[\begin{array}{ccc} \cos\theta & 0 & -\sin\theta \\ 0 & 1 & 0\\ \sin\theta & 0 & \cos\theta \end{array}\right]$$

In this framework, the rotation angle θ follows a uniform distribution U(0,2π). The global geometric transformation is applied via matrix multiplication, expressed as $P^{m}=P\cdot R_{y}\left(\theta \right)$. Furthermore, Gaussian noise is incorporated by perturbing the positions within the point cloud. This noise is typically described by a distribution Δ∼N(0,0.01), with a specified offset of 0.05 added to perturb point positions. Consequently, the modified point cloud is expressed as $P^{j}=P+\Delta$.

Jitter enhancement, as described in reference [25], introduces Gaussian noise or point cloud coordinates to simulate sensor noise or minor deformations. This technique boosts the model’s robustness against real-world noise data. Implementing jitter enhancement requires careful tuning of noise intensity, framework compatibility, and feature synchronization. In our approach, we set the noise intensity to 0.01 with a maximum absolute noise threshold of 0.05 to prevent excessive distortion of the point cloud.

We adopt a region-based masking strategy in our framework due to its strong alignment with the characteristics of real-world 3D scenes. Unlike random point masking, which discards isolated points and may fail to capture structural semantics, region masking occludes spatially contiguous subsets of the point cloud, thereby simulating realistic scenarios such as object occlusion, missing scans, or sensor noise. This strategy compels the network to reason over larger spatial contexts and infer semantics from surrounding structures, which is particularly beneficial in large-scale outdoor environments where contextual dependencies dominate. Moreover, region masking avoids excessively sparse supervision signals by preserving the overall distribution of labeled points, making it more effective than random masking in weakly supervised settings. Empirical studies further demonstrate that region masking leads to more robust feature learning and improves the model’s ability to generalize to unseen data.

**Figure 2:**
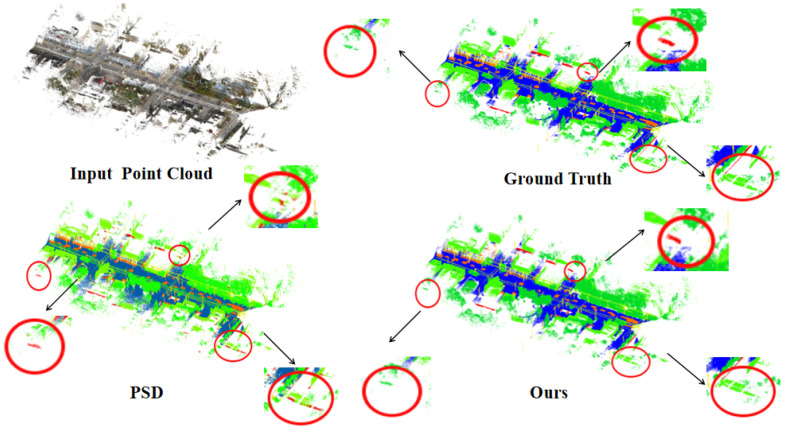
Qualitative comparison between PSD [12] and our qualitative results on the Toronto-3D dataset [26], using 0.1% labeling for training. Compared to PSD, our approach achieves comparable or even superior results, with improved segmentation accuracy in certain detailed regions.

This technique encourages the network to focus on global semantics rather than overfitting to local details by simulating sensor occlusion or partial object loss. The point cloud’s 3D space is first divided into a uniform grid of dimensions G×G×G. Each grid cell is defined as a cubic region centered at $\left(x_{c},y_{c},z_{c}\right)$ with a side length of $\Delta =\frac{r}{G}$, where *r* represents the grid corresponding to a point $P_{i}=\left(x_{i},y_{i},z_{i}\right)$, which is calculated as $\left(g_{x_{i}},g_{y_{i}},g_{z_{i}}\right)=\left(\frac{x_{i}}{r},\frac{y_{i}}{r},\frac{z_{i}}{r}\right)$. The point cloud is then subjected to regional sampling, wherein *K* regions are randomly selected from the $G^{3}$ grids, where *K* is signified as $K=p\cdot G^{3}$, and *p* implies the masking ratio. Experimental investigations conducted across various masking ratios indicate that the optimal training performance during the training process occurs at a masking ratio of 0.2, and we highlight our segmentation results compared to others with red circles. As shown in Figure 2. To prevent overfitting, the masking range is randomly adjusted throughout the training phase. This region masking consistently obscures the irregular and unordered point clouds, thereby helping the model extract meaningful contextual patterns.

In the preceding discussion, we introduced four data enhancement strategies, one of which is randomly chosen during point cloud data training. Real-world point clouds exhibit diverse attributes; for example, color is essential for distinguishing categories such as doors and windows. However, in classes with similar colors such as columns and walls, relying on color may hinder effective feature extraction. To address this issue, we introduce an attribute attention layer that adaptively learns weights for the input attributes, serving as a learnable transformation to handle the inherent diversity of point clouds. Specifically, the enhanced point cloud features are concatenated with the original ones to form a combined feature, *F*, which is then processed through an unbiased fully connected layer, producing a channel attention score S=W·F, where *W* indicates the learnable parameter matrix. The attention distribution is subsequently obtained through normalization via the Softmax function [27], expressed as A=Softmax(S). Ultimately, the perturbed point cloud is created by applying the learned feature weights.

### 3.3. The Dual-Branch Network Architecture

To enhance the model’s capacity for extracting contextual semantic features from point clouds, provide stronger supervisory signals with limited labeled data, and generate higher-quality segmentation results, this study proposes a dual-branch consistency learning network with an EMA teacher mechanism. The primary architecture is based on the RandLA-Net [3]. The overall structure of the network is shown in Figure 1. Each branch network follows an encoder–decoder architecture, where the encoder serves as the feature extractor for the point cloud data. The rationale for our architectural choices stems from three principles:

1. Diversity–Stability Trade-Off: The deeper student explores complex hypothesis spaces while the shallower teacher maintains stable targets.
2. Information Filtering Hypothesis: The deep–shallow parameter mapping filters out task-irrelevant features, retaining robust representations.
3. Temporal Smoothing as Regularization: Teacher weights represent temporally smoothed student behavior, preventing overfitting to recent batches.

The student network builds upon an enhanced hierarchical architecture that processes both original and augmented point clouds. The encoder integrates dilated residual blocks with local feature aggregation through relative position encoding and attention-based pooling. As point cloud P with N points progresses through the encoder layers, systematic downsampling reduces point count while expanding feature dimensions: $N\to \frac{N}{4}\to \frac{N}{16}\to \frac{N}{64}\to \frac{N}{256}\to \frac{N}{1024}$. Correspondingly, feature dimensions increase progressively from 8 to 512, capturing multi-scale contextual information while preserving structural details through skip connections.

The teacher network employs a streamlined three-layer encoder that processes only the original point cloud, with parameters updated via an exponential moving average (EMA) from the student network. This design provides stable supervisory signals while maintaining computational efficiency. The teacher branch undergoes similar dimensional transformations $\left(N\times 8\to \frac{N}{4}\times 32,\frac{N}{16}\times 256,\frac{N}{64}\times 512\right)$, enabling effective global context capture with reduced computational overhead.

The decoder processes features from the final encoder layer through a hierarchical upsampling path. At each stage, features from the preceding decoder layer are combined with corresponding encoder features via skip connections, after applying 1 × 1 convolutions for dimensional alignment. Nearest-neighbor interpolation restores spatial resolution using stored indices from the encoder’s downsampling operations. Feature fusion occurs through channel-wise concatenation followed by transpose convolution for enhanced feature learning. The final output is generated through 1 × 1 convolutional layers that produce high-level semantic segmentation logits.

The encoder of the branch network consists of three layers, each functioning similarly to those in the backbone network, aiming to effectively capture the global information inherent within the point cloud. After the decoder, the output features of the point cloud undergo dropout regularization [28], which enhances the model’s robustness by randomly masking neurons to help prevent overfitting. These features are then processed through a classification layer, where convolutional operations map them to the task-specific category space. This process produces prediction data for each category within the point cloud, and the argmax function [29] is applied to identify the index of the maximum value for segmentation purposes. Ultimately, the output comprises the predicted information for the point cloud.

In the decoder phase, both networks employ nearest interpolation with skip connections to recover spatial resolution. The student decoder integrates features from corresponding encoder layers through feature concatenation and transpose convolution operations, progressively restoring point cloud resolution while incorporating multi-scale contextual information. The teacher decoder follows a similar pattern but processes only the original point cloud features.

The architecture incorporates structural context modules with edge convolution to enhance local geometric awareness, and introduces a graph-structured Laplacian smoothing loss that enforces local prediction consistency by minimizing the symmetric KL divergence between the probability distributions of neighboring points. This multi-objective optimization framework integrates cross-entropy loss, Lovasz loss, Jensen–Shannon divergence [30], and contrastive regularization, thereby achieving both local smoothness and global semantic accuracy while ensuring robustness to noise and structural variations.

### 3.4. Structure-Relation Context (SR-Context) Module

To explicitly encode local geometric relationships and enhance the model’s awareness of point cloud structure, we introduce a Structure-Relation Context (SR-Context) module. This module operates directly on point-level features and is designed to capture context from the local neighborhood of each point.

Given the input point-wise features $F\in P^{N\times d}$, where *N* is the number of points and *d* is the feature dimension, the module first constructs a local graph using the *k*-nearest neighbors (*k*-NN) based on the point coordinates. For each point *i*, let N(*i*) denote its neighbor set. We then compute edge-aware features by applying an EdgeConv-style operation: $j\in N^{i}$.

(3) $$e_{ij}=MLP\left(f_{i}⊕\left(f_{j}-f_{i}\right)\right),$$

where ⊕ denotes concatenation, $f_{i}$ and $f_{j}$ are features of point *i* and its neighbor *j*, and $e_{ij}$ is the resulting edge feature. The edge features are aggregated via a channel-wise attentive pooling scheme:

(4) $$a_{ij}=\sigma (W_{a}e_{ij})),$$

where σ is the sigmoid function, $W_{a}$ is a learnable weight matrix, ⊙ denotes element-wise multiplication, and max(·) performs channel-wise max pooling.

Finally, the original feature and the structure-aware feature are concatenated and passed through a lightweight projection layer to obtain the enhanced output:

(5) $$\;F^{\prime }=Conv_{1\times 1}\left(F⊕F_{0}\;\right)),$$

where $F_{0}$ = [$F_{∦}\ldots F_{N}{]}^{T}$. The SR-Context module is lightweight and differentiable, allowing it to be inserted at multiple stages of the network. It provides a strong geometric inductive bias that helps the model better distinguish between semantically similar but structurally different objects (e.g., chairs vs. tables), which is particularly beneficial in weakly supervised settings where labeled data is scarce.

### 3.5. Loss Function Design

The loss function in our proposed framework is designed as a comprehensive multi-objective optimization strategy that integrates multiple complementary components to enhance segmentation performance and ensure consistency across the dual-branch architecture.

The cross-entropy loss (Lce) measures the divergence between predicted probability distributions and ground truth labels, with class-weighted adjustments to address category imbalance in point cloud data. Simultaneously, the Lovasz-softmax loss (Llovasz) directly optimizes the intersection-over-union (IoU) metric, effectively handling the non-convex and combinatorial nature of segmentation evaluation while being particularly beneficial for imbalanced class distributions.

Beyond these fundamental segmentation losses, we introduce several consistency and regularization terms: JS divergence loss ($L_{JS}$) enforces prediction consistency between the student network’s original and augmented views, as well as between student and teacher network outputs.

Contrastive regularization loss ($L_{CR}$) aligns feature embeddings by maximizing agreement between semantically similar points while separating dissimilar ones.

Graph Laplacian smoothing loss ($L_{Laplacian}$) explicitly enforces local consistency in the prediction space by minimizing symmetric KL divergence between neighboring points’ probability distributions.

The complete optimization objective is formulated as a weighted combination:

(6) $$L_{total}=L_{ce}+L_{lovasz}+\lambda_{\mathrm{JS}\;}L_{\mathrm{JS}}\;+\lambda_{\mathrm{CR}}L_{\mathrm{CR}}+\lambda_{L}\lambda_{\mathrm{Laplacian}}$$

where $L\in P^{N\times C}$ indicates the tensor of predicted labels; *C* implies the number of categories; and $Y\in P^{N}$ signifies the actual labels. Additionally, let *K* refer to the number of labels present in the point cloud. Consequently, the associated weighted CEloss can be expressed as follows:

(7) $$L_{ce1}=-\frac{1}{2(2K)}\sum_{i=1}^{2K}\sum_{j=1}^{2K}\left(a_{ij}\log p_{ij}+\left(1-a_{ij}\right)\log\left(1-p_{ij}\right)\right)$$

(8) $$L_{ce2}=-\frac{1}{2(2K)}\sum_{i=1}^{2K}\sum_{j=1}^{2K}\left(b_{ij}\log q_{ij}+\left(1-b_{ij}\right)\log\left(1-q_{ij}\right)\right)$$

where *i* and *j* imply distinct points within a point cloud and $p_{ij}$ and $q_{ij}$ signify the categorical relationship between points *i* and *j*, respectively. Additionally, the variables $a_{ij}$ and $b_{ij}$ are employed to represent the semantic relationship between points *i* and *j*. The Lovasz loss function [31], as referenced in the literature, is designed to optimize the IoU metric and is frequently employed in segmentation tasks. Specifically,

(9) $$L_{\mathrm{lovasz}\;}=\frac{1}{C}\sum_{C=1}^{C}\Delta_{L}\left(P_{c},y_{c}\right)$$

where $\Delta_{L}$ indicates the convex substitution function associated with the Jaccard exponential error for category *C*. An illustration of a convex substitution function is provided, where $P_{c}$ signifies the predicted labeling information and $y_{c}$ signifies the true labeling information.

### 3.6. Consistency Loss Function Design

We employ a comprehensive consistency regularization framework using Jensen–Shannon divergence [30] to enforce prediction alignment between the student and teacher networks, as well as between original and augmented point cloud views processed by the student network. This includes augmentation consistency loss between the student’s predictions for original and augmented data:

(10) $$LJS-aug=\frac{1}{N}\sum {i=1}^{N}\left[D_{JS}\left(P_{s}^{orig}\|P_{s}^{aug}\right)+D_{JS}\left(P_{s}^{aug}\|P_{s}^{orig}\right)\right]$$

and teacher–student consistency loss between the student and EMA teacher predictions:

(11) $$LJS-tea=\frac{1}{N}\sum {i=1}^{N}\left[D_{JS}\left(P_{s}^{orig}\|P_{t}\right)+D_{JS}\left(P_{t}\|P_{s}^{orig}\right)\right]$$

where $P_{s}^{orig}$ and $P_{s}^{orig}$ represent the student network’s predicted probability distributions for original and augmented point clouds, respectively, and $P_{t}$ denotes the teacher network’s predictions. Additionally, we incorporate contrastive regularization loss to align feature embeddings:

(12) $$LCR=-\frac{1}{N}\sum {i=1}^{N}\log\frac{\exp(\mathrm{sim}\left(ei^{1},ei^{2}\right)/\tau )}{\sum {j=1}^{N}\left[j\neq i\right]\exp\left(\mathrm{sim}\left(e_{i}^{1},e_{j}^{2}\right)/\tau \right)}$$

where $e_{i}^{1}$ and $e_{i}^{2}$ represent feature embeddings from different augmentations of the same point, and τ is a temperature parameter. The graph Laplacian smoothing loss further enforces local consistency:

(13) $$Llaplacian=\frac{1}{NK}\sum {i=1}^{N}\sum_{k=1}^{K}\left[D_{KL}\left(P_{i}\|P_{N(i,k)}\right)+D_{KL}\left(P_{N(i,k)}\|P_{i}\right)\right]$$

where $P_{N(i,k)}$ represents the prediction of the k-th neighbor of point i. The complete optimization objective integrates all components:

(14) $$Ltotal=Lseg+\lambda_{JS}\left(LJS-aug+LJS-tea\right)+\lambda_{CR}LCR+\lambda LL_{Laplacian}$$

where Lseg=Lce+$L_{Laplacian}$ combines weighted cross-entropy and Lovasz losses for semantic segmentation, and $\lambda_{JS}$, $\lambda_{CR}$, and $\lambda_{L}$ are balancing parameters that control the relative importance of each consistency term.

## 4. Experiments

### 4.1. Dataset and Evaluated Metrics

This study utilizes two benchmark datasets: the S3DIS dataset [31] and the Toronto-3D dataset [26]. RandLA-Net [3] is employed as the backbone network, with no pretraining conducted on the test data, thereby guaranteeing that the model has not been exposed to real data and avoiding any risk of data leakage. The S3DIS dataset [31] comprises six large-scale indoor regions with a total of 271 rooms and includes six attributes per point—namely, the XYZ coordinates and RGB color values. This dataset is widely used for evaluating semantic segmentation performance in indoor environments. In contrast, the Toronto-3D dataset [26] represents a significant outdoor point cloud dataset collected using an MLS system in Toronto, Canada. It spans approximately 1 km of roadway and consists of roughly 78.3 million points. This dataset is characterized by ten attributes that are categorized into eight distinct object classes.

We selected the RandLA-Net [3] as our backbone network due to its computational efficiency and strong performance on large-scale point clouds, making it a suitable foundation for our dual-branch architecture.

Hyperparameter Settings and Justification: The model was optimized using Adam with an initial learning rate of $1\times 10^{-3}$. This rate was determined through a grid search on a held-out validation set, which showed that it provided stable convergence. We employed a step decay schedule, reducing the learning rate by a factor of 0.1 at 60 and 80 epochs, a common practice to refine learning in the final training stages. The input point clouds were subsampled to 40,960 points using a combination of random and furthest point sampling (FPS) to ensure a representative spatial distribution. A training batch size of 4 was used, constrained by GPU memory (NVIDIA RTX 4090), while a larger validation/test batch size of 12 was used for efficient evaluation. All models were trained for 100 epochs, sufficient for convergence as observed from plateauing validation metrics.

Key Framework-Specific Parameters: For the exponential moving average (EMA) teacher update, a decay rate of 0.999 was adopted. This follows the standard in mean teacher paradigms [13] to ensure a stable teacher that evolves smoothly, providing consistent pseudo-labels. The regionwise masking ratio for structural augmentation was set to R = 0.2. This value was chosen empirically, as it introduces meaningful geometric perturbation without excessively corrupting the global structure of the point cloud (see Section 4.4 for ablation experiments). Consistency between branches was enforced using the Jensen–Shannon Divergence [30], and the Laplacian smoothing weight was set to 0.10, balancing local structural regularization with the primary task loss.

Sensitivity Analysis: To validate the robustness of our critical hyperparameters, we conducted a sensitivity analysis on the EMA decay rate and the mask ratio *R*. The results (detailed in Section 4.4) show that performance is robust within a reasonable range around our chosen values (e.g., R ∈[0.15,0.7]), confirming that our method does not require extreme fine-tuning.

All experiments were conducted on an NVIDIA GeForce RTX 4090 GPU. A complete training cycle required approximately 8 h. Model performance was evaluated using the mean intersection over union (mIoU, %) as the primary metric. As summarized in Table 1, our method demonstrates superior training and inference efficiency compared to recent semi-supervised counterparts under the same 0.1% labeling budget. Notably, our model requires only 8 h for training, which is 3 faster than PSD [12] and even shorter than the fully supervised RandLA-Net [3]. This accelerated convergence is attributed to our multi-hierarchical consistency losses, which provide dense and effective supervisory signals. During inference, our framework processes a point cloud in 40 ms, achieving the fastest speed among semi-supervised methods and closely approaching the efficiency of RandLA-Net. Moreover, with only 2.08 M parameters, our model maintains a compact architecture. These results collectively indicate that our approach not only reduces annotation cost but also offers significant advantages in computational efficiency, making it highly practical for real-world applications.

### 4.2. Quantitative Results on S3DIS

Table 2 presents the quantitative evaluation results for Region 5 of the S3DIS dataset [31], offering a comparative analysis of our method against alternative methodologies. The evaluation is based on the IoU metrics, specifically mean IoU (mIoU, %) and IoU per class (%), with the highest results achieved under weak labeling conditions highlighted in bold. To maintain a rigorous comparative framework, our methodology was evaluated using the same parameters as previous studies. Notably, in the weakly supervised task, we achieved significant results at annotation levels of 0.1% and 0.02%, respectively. Specifically, at the 0.1% labeled points threshold, our approach outperformed the CPCM [7] method by 2.26% in mIoU and showed improvements of 9.82%, 2.61%, and 10.1% in the categories of window, floor, and chair, respectively.

**Figure 3:**
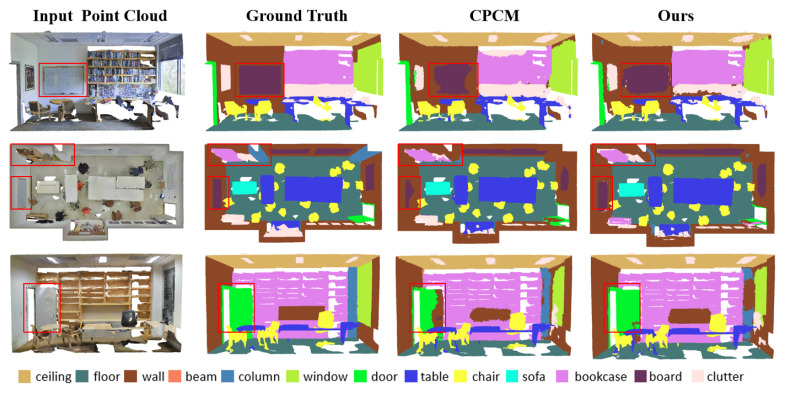
Comparison of our method with existing ones on S3DIS dataset indoor scene, We use red boxes to compare the benchmark results and our results. It can be seen that our segmentation results are superior to the benchmark and other results. Ref. [31] using 1% labeling scale.

To intuitively understand the effectiveness of our segmentation approach, we provide visual comparisons in Figure 3. First, we observe that our dual-branch network demonstrates superior performance in understanding semantic categories with varying appearances (e.g., wall, Row 1) and handling large geometric objects (e.g., bookcase, Row 2). Furthermore, we recognize its remarkable capability in distinguishing between geometrically and visually similar categories (e.g., door, Row 3).

### 4.3. Quantitative Results on Toronto-3D

We performed a thorough evaluation of the outdoor scene Toronto-3D dataset [26], testing our method at labeling rates of 0.1%, 0.02%, 1%, and 10%. As shown in Table 3, with a uniform mask percentage of 20%, our approach exceeded SQN [34] by 0.2% at the 0.1% labeling level and RandLA-Net [3] by 0.02% under full supervision. These findings highlight the efficacy of our segmentation capabilities and the robust generalization potential of the dual-stream network model.

The original class proportions are as follows: Road points dominate the dataset at around 53.4%, followed by Building at 24.4%, Natural at 8.4%, Car at 5.0%, Road marking at 2.3%, Pole at 1.1%, Utility Line at 0.8%, Fence at 0.5%, and Unclassified at 3.9%. The distribution remains consistent after random sampling.

**Figure 4:**
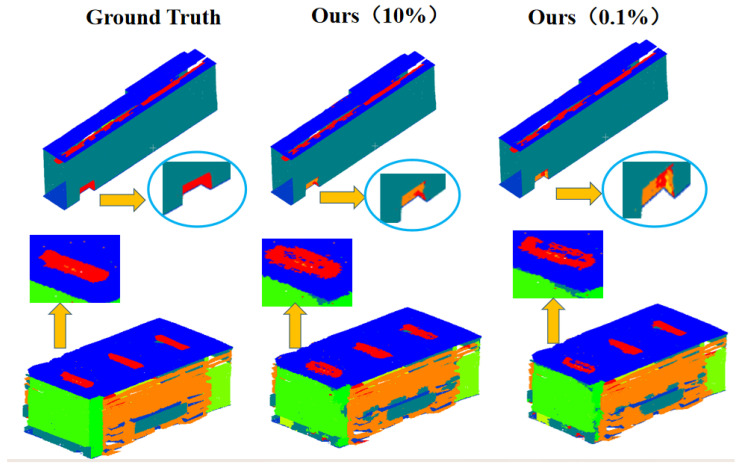
Visualization of our segmentation results at various labeling scales on the S3DIS dataset [31]. It can be seen that our segmentation results are more complete even with 0.1% weakly labeled annotations.

We also visualized results on the Toronto-3D dataset [26], as depicted in Figure 2. Compared to the PSD [12], our method demonstrates superior details, particularly in the representation of streets and vehicles, and the classification of residential structures, as highlighted by the red markers. Additionally, we compared our results with those of RandLA-Net [3] across various scenes, as illustrated in Figure 4. Using the S3DIS dataset [31], we visualized indoor scenes with differently labeled point clouds. Even at a low labeling rate of 0.1%, our segmentation results closely approximate the ground truth, demonstrating superior smoothness and a more complete external profile.

Figure 4 illustrates the semantic segmentation performance on the S3DIS dataset [31] under varying annotation rates (10%, 0.1%, and ground truth). Compared to the ground truth, our model yields remarkably complete and coherent segmentation maps even when only 0.1% of points are annotated, closely approximating the full-supervision results. The zoomed-in regions enclosed by green circles highlight the capability of our method to preserve fine boundaries and highlight the contrasting pattern with yellow arrows;object contours (e.g., building foundations) remain sharp and continuous, avoiding the fragmentation or erosion commonly observed in baseline methods under weak supervision. Internal consistency is also maintained, as large planar regions (e.g., window surfaces) are segmented smoothly without spurious noisy predictions. This can be attributed to the Laplacian smoothing loss and contrastive regularization, which explicitly enforce local prediction consistency and enhance feature discrimination across semantically similar regions.

**Figure 5:**
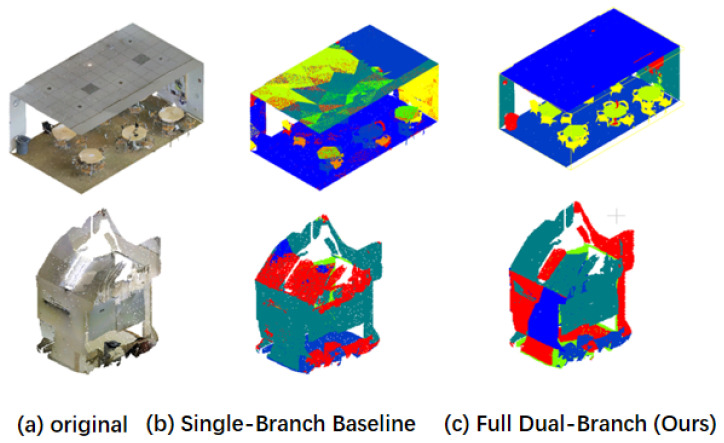
Comparative analysis of network architectures. As demonstrated in the qualitative comparison, our dual-branch network exhibits significantly superior visualization results in numerous details compared to the single-branch counterpart. Among them, the houses above are marked in blue, and the roofs below are marked in red and green. This improvement is particularly evident in objects with complex appearances and structures, such as windows and chairs, where our architecture achieves more precise boundary delineation and semantically consistent segmentation.

Additionally, Figure 5 demonstrates that our dual-branch network clearly outperforms the single-branch network in visualizing many details. Fine structures, such as table legs and window frames, are preserved thanks to the structure-aware enhancement and the SR-Context module that explicitly encodes local geometric information. This architecture enables more precise boundary rendering and semantically consistent segmentation.

These visual improvements are direct manifestations of our core technical contributions. The consistency enforced between the student and teacher branches through multi-hierarchical losses (JS divergence and contrastive regularization) propagates supervisory signals effectively, mitigating overfitting to the sparse annotations. Specifically, the Laplacian smoothing loss explicitly promotes local prediction homogeneity, while the Structure Relation Context (SR-Context) module enhances the model’s sensitivity to geometric primitives, leading to more structurally plausible segmentations. This qualitative evidence aligns with and substantiates the quantitative gains reported in Table 4, confirming that our dual-branch design and associated regularization strategies are pivotal for achieving robust, high-fidelity segmentation in low-label regimes.

### 4.4. Ablation Experiments

Our point cloud segmentation framework leverages a dual-branch consistency learning architecture with an EMA teacher mechanism, whose effectiveness is validated through comprehensive comparisons with conventional single-branch networks. As shown in Table 5, under full supervision, our model achieves 4.19% higher segmentation accuracy than AADNet [38] and outperforms MIL [5] by 2.09%. More significantly, under the weakly supervised setting with only 0.01% labeled data.

**Figure 6:**
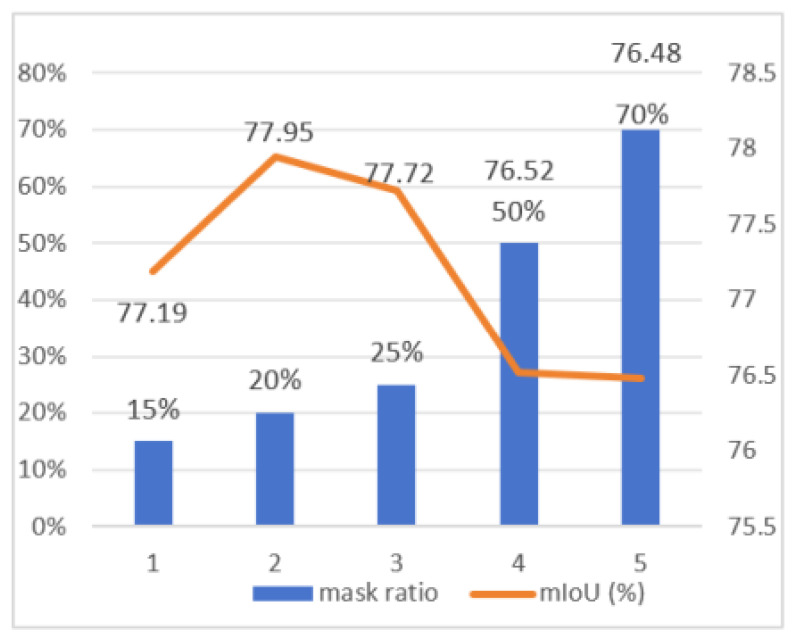
Segmentation results across different mask scales on the Toronto-3D dataset [26], using 0.1% labeling.

We further evaluated the impact of different mask scales on the Toronto-3D dataset [26] using a labeling rate of 0.1%, as detailed in Figure 6. The results show that segmentation accuracy peaks at an R of 20%, with a gradual decline beyond 50%. These experiments were conducted on the L002 scene of the Toronto-3D dataset [26], with evaluations performed on a dedicated point set. The experimental results demonstrate that our methodology effectively captures contextual similarities, enabling the network to develop a more accurate and coherent understanding of spatial relationships. Furthermore, the approach improves feature discrimination by effectively establishing relationships between labeled and unlabeled points.

We conducted comprehensive ablation experiments to evaluate the effectiveness of our proposed dual-branch architecture combined with the EMA teacher mechanism in Table 4. All experiments were carried out on the S3DIS dataset (Area 5), using only 0.1% of the labeled data to simulate a scenario of weak supervision. The baseline model adopted a single-branch RandLA-Net [3] architecture and used standard cross-entropy and Lovasz loss. The basic dual-branch architecture without EMA achieves an 8.3% mIoU improvement over the single-branch baseline, demonstrating the benefit of processing both original and augmented point clouds through separate pathways. Incorporating exponential moving average updates for the teacher branch provides an additional 3.1% performance gain.

The ablation experiments conclusively demonstrate that our dual-branch architecture with the EMA teacher mechanism provides substantial performance improvements in weakly supervised point cloud segmentation. The EMA update strategy proves crucial to maintaining stable supervision, while parameter sharing optimization ensures computational efficiency without compromising accuracy. These findings validate our architectural design choices and highlight the importance of consistent teacher–student collaboration in limited supervision scenarios.

Table 6 presents a systematic ablation study on the Toronto-3D dataset [26], quantifying the contribution of individual components common in consistency learning frameworks. The experiment is structured as an incremental addition of key components: starting from a basic cross-entropy (CE) baseline (MS-PCNN [36]), we successively add perturbation consistency (KL Perturb in KPFCNN [35]), teacher consistency (KL Teacher in RandLA-Net [3]), contrastive regularization (CR in TGNet [37]), and finally Laplacian smoothing (Lap in CPCM [7]).

The results demonstrate a clear cumulative benefit, with each added component improving the mIoU: +4.91% (KL Perturb), +1.85% (KL Teacher), +5.46% (CR), and +3.21% (Lap). This empirically validates that all these loss terms are necessary for achieving state-of-the-art performance, as they address complementary aspects of semi-supervised learning: data invariance, stable pseudo-labeling, feature discrimination, and structural coherence.

Our proposed method, which integrates all these components within a novel heterogeneous dual-branch EMA framework, achieves the best performance of 80.00% mIoU, outperforming the previous best component-aggregating method (CPCM [7]) by 3.65%. This significant margin underscores that our contribution lies not merely in the aggregation of these components, but in their synergistic integration through a dedicated architecture (e.g., cross-architectural EMA updates, SR-Context module for feature refinement), which enables more effective and robust consistency learning.

## 5. Conclusions

This study presents a dual-branch consistency learning framework with an EMA teacher mechanism for weakly supervised point cloud semantic segmentation. By incorporating supplementary supervisory cues for unlabeled data, the model surpasses conventional single-layer networks and improves its focus on the global characteristics of point clouds. Consequently, the proposed network consistently delivers accurate and reliable point cloud segmentation across various states. Extensive experimental results indicate that our method holds strong promise for weakly supervised semantic segmentation of large-scale point clouds. Extensive experimental results demonstrate that our method achieves competitive performance in weakly supervised point cloud segmentation while maintaining computational efficiency. Future work will focus on extending the dual-branch consistency learning framework to weakly supervised point cloud detection and instance segmentation tasks, exploring more efficient graph construction methods for Laplacian smoothing, and investigating adaptive weighting strategies for the multi-objective loss function to further enhance performance across diverse 3D scenes.

### 5.1. Discussion and Limitations

Although our proposed framework demonstrates strong performance on established indoor (S3DIS) and outdoor (SemanticKITTI/Toronto-3D) benchmarks in extreme low-label settings, we acknowledge several limitations that point to valuable future work.

Our evaluation is primarily within the dataset; cross-domain generalization (e.g., from driving to medical scans) remains challenging due to shifts in density, scale, and semantics. Future work requires rigorous cross-dataset benchmarks (e.g., aerial LiDAR) and integration of domain adaptation into our framework. Additionally, while efficient, the dual-branch design increases training cost, and performance is backbone-dependent. Future directions include architectural refinements for greater efficiency and testing on broader 3D benchmarks.

### 5.2. Limitations and Trade-Offs

A core trade-off exists between high accuracy under extreme label scarcity and increased training cost, a deliberate choice to maximize learning from minimal labels. Performance also depends on the backbone network’s capacity; while our design is modular, we used RandLA-Net for its efficiency. Hyperparameter tuning is required, but sensitivity analysis confirms robustness within a practical range. Finally, like most data-driven methods, optimal performance assumes similar training and test distributions; generalization across vastly different domains remains challenging and may require dedicated adaptation techniques.

### 5.3. Future Work

Building upon the identified limitations and trade-offs, several promising directions emerge for future research: (1) architectural distillation into a single efficient network for deployment; (2) exploring integration with more powerful backbones (e.g., transformers) to boost performance; (3) incorporating domain adaptation techniques to enhance cross-domain robustness; (4) extensive validation on broader 3D benchmarks (e.g., aerial LiDAR, medical point clouds). 

## Figures and Tables

**Table 1:** Efficiency comparison on the S3DIS dataset [31] (Area 5). All experiments were conducted on a single NVIDIA GeForce RTX 4090 GPU with 512 GB memory. Inference time is measured per point cloud with 40,960 points. The one with the best performance is shown in bold. Note: GPU memory is the peak consumption during inference for a standard point cloud. Training time is the total wall-clock time to achieve the reported performance.

Method	Setting	Params (M)	Inference Time (ms)	Training Time (h)
RandLA-Net [3]	Fully	1.24	**29.6**	10
PointMatch [32]	0.1%	**4.32**	45.2	12
PSD [12]	0.1%	2.15	67.3	24
Dual-Branch (Ours)	0.1%	2.08	40	**8**

**Table 2:** Quantitative results of various methods on Region 5 of the S3DIS dataset [31]. Bold represents the best result in weakly labeled settings and fully labeled settings.

Method	Setting	mIoU (%)	Ceiling	Floor	Wall	Beam	Column	Window	Door	Table	Chair	Sofa	Bookcase	Board	Clutter
PointNet [2]		41.1	88.8	**97.3**	69.8	0.0	4	46.3	10.8	58.9	52.6	5.9	40.3	26.4	33.2
RandLA-Net [3]	Fully	**65.1**	92.3	97.1	80.6	0.0	18.4	**61.5**	**68.1**	77.9	**86.2**	**71.5**	**71**	69	53
PSD [12]		63	**92.4**	96.7	**80.7**	0.0	**32.3**	55.3	43.3	**78.2**	85.8	71.1	70.6	59.2	52.3
Xu and Lee [25]		48	90.9	97.3	74.8	0.0	8.4	49.3	27.3	69	71.7	16.5	53.2	23.3	42.8
II Model [33]	10%	46.3	91.8	97.1	73.8	0.0	5.1	42	19.6	66.7	70.7	19.4	47.9	30.6	41.3
MT [13]		47.9	92.2	96.8	74.1	0.0	10.4	46.2	17.7	67	67.2	24.1	50.2	30.7	**42.2**
Zhang et al. [22]		61.8	91.5	96.9	80.6	0.0	18.2	58.1	47.2	75.4	**88.7**	62.2	68.9	65	50.6
PSD [12]	1%	63.5	**93.3**	**97.7**	81.7	0.0	**30.8**	63.2	62.5	**78.7**	84.1	63.1	70.4	58.4	53.2
HybridCR [20]		**65.3**	92.5	93.9	**82.6**	0.0	24.2	**64.4**	**63.2**	78.3	81.7	**69.0**	**74.4**	**68.2**	**56.5**
RandLA-Net [3]		52.9	89.9	95.9	75.3	0.0	7.5	52.4	26.5	62.2	73.5	49.1	60.2	49.3	45.1
SQN [34]	0.1%	61.4	91.7	95.6	78.7	0.0	24.2	55.9	63.1	62.9	70.5	67.8	60.7	56.1	50.6
CPCM [7]		66.3	91.4	95.5	82.0	0.0	30.8	54.1	70.1	87.6	79.4	70.0	67.0	77.8	56.6
Ours		**68.56**	**93.40**	**98.11**	**85.09**	**0.02**	**32.75**	**63.92**	63.67	81.50	**89.50**	**77.34**	**73.04**	74.67	**58.27**
MIL [5]		52.1	89.9	95.5	74.8	0.2	19.2	55.1	23.1	76.3	64.7	62.6	27.8	26.8	44.8
CPCM [7]	0.02%	62.3	92.6	95.6	79.4	0.0	17.8	49.3	59.4	85.7	75.6	69.1	60.7	68.2	55.8
Ours		**63**	**92.93**	95.55	**80.65**	0.0	**24.99**	**51.11**	54.61	79.87	72.3	**71.09**	**65.9**	65.49	**58.3**

**Table 3:** Quantitative results of various methods on the Toronto-3D dataset, The one with the best performance is shown in bold [26].

Method	Setting	mIoU (%)	Road	Rd mrk.	Natural	Building	Util. Line	Pole	Car	Fence
PointNet++ [14]	Fully	41.81	89.27	0	69.06	54.16	43.78	23.3	52	2.95
KPFCNN [35]		**69.11**	**94.62**	0.06	**96.07**	**91.51**	**87.68**	**81.56**	85.66	15.72
MS-PCNN [36]		65.89	93.84	**3.83**	93.46	82.59	67.8	71.95	**91.12**	**22.5**
TGNet [37]		61.34	93.54	0	90.83	81.57	65.26	62.98	88.73	7.85
RandLA-Net [3]	10%	**81.88**	96.69	64.1	**96.85**	**94.14**	**88.03**	77.48	93.21	**44.53**
Ours		80.88	**97.79**	**72.62**	95.91	92.61	86.56	**80.54**	**95.27**	25.8
Ours	0.1%	**77.95**	95.44	56.27	**95.90**	**92.80**	**85.81**	**87.97**	**90.90**	27.53
SQN [34]		77.75	**96.69**	**65.67**	94.58	91.34	83.36	70.59	88.87	**30.91**

**Table 4:** Ablation study on dual-branch architectures on S3DIS dataset [31], using 0.1% label. The one with the best performance is shown in bold.

Configuration	Student Branch	Teacher Branch	EMA Update	mIoU (%)
Single-Branch Baseline	🗸	x	x	52.9
Dual-Branch w/o EMA	🗸	🗸	🗸	61.2
Dual-Branch w/o EMA	🗸	🗸	🗸	64.3
Full Dual-Branch (Ours)	🗸	🗸	🗸	**68.56**

**Table 5:** Comparison of experimental results for various structures in S3DIS dataset [31], The one with the best performance is shown in bold.

Method	Setting	mIoU (%)
PointNet++ [14]	Fully	41.81
TGNet [37]	Fully	61.34
PVT2 [39]	Fully	71.6
SQN [34]	0.1%	61.4
PointMatch [32]	0.1%	63.4
AADNet [38]	0.1%	67.2
Dual-Branch (Ours)	0.1%	**68.56**
PointMatch [32]	0.01%	59.9
AADNet [38]	0.01%	60.8
MIL [5]	0.01%	62.9
Dual-Branch (Ours)	0.01%	**64.99**

**Table 6:** Ablation study on loss components for semi-supervised point cloud segmentation on Toronto-3D dataset [26]. The one with the best performance is shown in bold.

Method	CE	KL (Perturb)	KL (Teacher)	CR	Lap	mIoU (%)	Δ mIoU
MS-PCNN [36]	🗸					61.34	–
KPFCNN [35]	🗸	🗸				66.25	**+4.91**
RandLA-Net [3]	🗸	🗸	🗸			68.10	**+1.85**
TGNet [37]	🗸	🗸	🗸	🗸		73.56	**+5.46**
CPCM [7]	🗸	🗸	🗸	🗸	🗸	76.35	**+3.21**
Ours	🗸	🗸	🗸	🗸	🗸	**80.00**	**+3.65**

## Data Availability

The experiments in this study were conducted on publicly available datasets, including S3DIS, ScanNet and Toronto-3D dataset, These datasets are openly accessible at [https://github.com/ss841111/S3DISdianyunshujujiwangpanlianjiefenxiang GitHub-ss841111/S3DISdianyunshujujiwangpanlianjiefenxiang, https://github.com/ScanNet/ScanNet GitHub-ScanNet/ScanNet and https://github.com/WeikaiTan/Toronto-3D GitHub-WeikaiTan/ Toronto-3D: A Large-scale Mobile LiDAR Dataset for Semantic Segmentation of Urban Roadways]. All accessed on 25 October 2025.
